# Inference of the Oxidative Stress Network in *Anopheles stephensi* upon *Plasmodium* Infection

**DOI:** 10.1371/journal.pone.0114461

**Published:** 2014-12-04

**Authors:** Jatin Shrinet, Umesh Kumar Nandal, Tridibes Adak, Raj K. Bhatnagar, Sujatha Sunil

**Affiliations:** 1 International Centre for Genetic Engineering and Biotechnology, New Delhi, India; 2 Bioinformatics Laboratory, Department of Clinical Epidemiology, Biostatistics and Bioinformatics, Academic Medical Center, Amsterdam, the Netherlands; 3 National Institute of Malaria Research, New Delhi, India; Kansas State University, United States of America

## Abstract

Ookinete invasion of *Anopheles* midgut is a critical step for malaria transmission; the parasite numbers drop drastically and practically reach a minimum during the parasite's whole life cycle. At this stage, the parasite as well as the vector undergoes immense oxidative stress. Thereafter, the vector undergoes oxidative stress at different time points as the parasite invades its tissues during the parasite development. The present study was undertaken to reconstruct the network of differentially expressed genes involved in oxidative stress in *Anopheles stephensi* during *Plasmodium* development and maturation in the midgut. Using high throughput next generation sequencing methods, we generated the transcriptome of the *An. stephensi* midgut during *Plasmodium vinckei petteri* oocyst invasion of the midgut epithelium. Further, we utilized large datasets available on public domain on *Anopheles* during *Plasmodium* ookinete invasion and *Drosophila* datasets and arrived upon clusters of genes that may play a role in oxidative stress. Finally, we used support vector machines for the functional prediction of the un-annotated genes of *An. stephensi*. Integrating the results from all the different data analyses, we identified a total of 516 genes that were involved in oxidative stress in *An. stephensi* during *Plasmodium* development. The significantly regulated genes were further extracted from this gene cluster and used to infer an oxidative stress network of *An. stephensi*. Using system biology approaches, we have been able to ascertain the role of several putative genes in *An. stephensi* with respect to oxidative stress. Further experimental validations of these genes are underway.

## Introduction

Maintenance of redox homeostasis is critical for proper functioning of cellular processes and disruption of this prooxidant-antioxidant balance in a cell results in oxidative stress. Oxidative stress may be caused by the normal functioning of the cell (mitochondrial respiration) or as an immune response to pathogens [1], [2] and is manifested by an increase in reactive oxygen species (ROS) and reactive nitrogen species (RNS) in the cells. These reactive species are capable of modifying DNA and proteins, inactivating biological activity and causing oxidative injury [3], [4]

Several studies have established that generation of ROS can be endogenous due to the leakage of activated oxygen from mitochondria during oxidative phosphorylation, peroxisomes, and activated inflammatory cells [5] or exogenous inflammatory cytokines, pathogens, and metals [6], [7]. ROS are toxic to cells and there are several detoxifying mechanisms that are employed by the cell to prevent oxidative damage.

*Plasmodium*, the causative agent of malaria, leads a complex life cycle, alternating between two hosts, vertebrate and invertebrate, with diverse environmental and physiological regimens. Further, within these two hosts, the parasite also exists as intra- and extracellular forms thereby being exposed to extreme surroundings. Several studies have revealed that *Plasmodium* undergoes immense oxidative stress during their erythrocyte cycle, considering that they live in a pro-oxidant environment in the red blood cells that contains oxygen and iron [8]–[10]. Recent studies have focused on targeting the Plasmodial redox system for anti-malarial therapy [11]. Several drugs have been developed to disrupt the mechanism and balance of ROS and RNS molecules, by targeting the enzymes of the parasite responsible for maintaining the redox balance [12]. During the mosquito cycle, the parasite undergoes tremendous oxidative stress. It can be rightly said that one of the major bottleneck in the parasite life cycle is the dwindling of its numbers during oocyst development in the mosquito stage [13]. However, it has been shown that *Plasmodium* overcomes this obstacle by using its defense mechanisms to protect against oxidative damage [10], [14], [15].

Just as in the case of *Plasmodium*, its vector, *Anopheles* also undergoes tremendous oxidative stress due to the high proliferative rate of the parasite and invasion of several of its organs by the parasite. The zygote transforms into motile ookinetes within 24 hours of ingestion of an infected blood meal and invades the mosquito midgut epithelium. Once inside, the ookinete develops into the oocyst between the basal lamina and the midgut epithelium. Upon maturity, the oocyst produces thousands of sporozoites that are released from the midgut into the hemocoel and finally reach the salivary glands. Here, they invade the salivary glands and mature to form the salivary gland derived sporozoites that are ready to infect the host during the next mosquito bite. During each of the invasion process and subsequent increase in parasite numbers, the mosquito undergoes extreme oxidative stress and several of the signaling pathways and innate immunity pathways are activated to protect the mosquito [16]–[20].

In the post-omics era, it is becoming clear that integration of genome-scale technologies provide better tools for understanding biological function [21]. Any cellular function is a dynamic interaction of several proteins to enforce a highly sensitive and a regulated system. A ‘single gene’-‘single function’ approach is fast being replaced by interaction networks for evaluating the intricacies involved in complex conditions like pathogen infection [22]–[24].

We have undertaken the present study to elaborate perturbations in the redox system of *An. stephensi* during successive stages of the development and maturation of *Plasmodium vinckei petteri*. Using next generation sequencing platforms, we obtained the transcriptome of the midgut of *An. stephensi* during *P. vinckei petteri* oocyst stage. We identified those transcripts that were differentially expressed and evaluated the dynamics of the *An. stephensi* redox system during oocyst development. Using Support Vector Machines (SVM) we classified unannotated genes of the transcriptome dataset into oxidative stress pathways. Additionally, we identified microarray datasets from public databases that studied *An. gambiae* during *Plasmodium* development, and arrived upon the set of *An. gambiae* genes involved specifically in oxidative stress during *Plasmodium* midgut invasion. Using all the above information, we inferred an almost complete network of the oxidative stress of *An. stephensi* during *Plasmodium* invasion.

## Materials and Methods

### Ethics statement

Animal experiments were performed in accordance with National animal ethics guidelines of the Government of India after approval by Institutional Animals Ethics Committees of International Centre for Genetic Engineering & Biotechnology, New Delhi (Permit number: ICGEB/AH/2011/01/IR-8).

### Mosquito rearing and *Plasmodium vinckei petteri* infection

*Anopheles stephensi* were reared at 28–30°C and humidity maintained at 70–75%. Mosquitoes were maintained by feeding with raisin soaked in 2% sterile glucose solution and water. 4–5 days old female mosquitoes were fed on *P. vinckei petteri* 279 BY (gametocytemia  = ∼0.05%) infected mice. Midguts from the infected mosquitoes were dissected on 5th day post infection and checked for the presence of oocysts for the confirmation of infection.

### *Anopheles stephensi* sample collection and RNA isolation

*Anopheles stephensi* midgut samples were collected from three different stages, namely, sugar fed (PVpSF), blood fed (PVpBF5D) and blood fed 5 days post *P. vinckei petteri* infection (PVpiBF5d). In case of infection and blood feed, around 150–200 *An. stephensi* mosquitoes were fed on *P. vinckei petteri* 279 BY (gametocytemia  = ∼0.05%) infected mice. Fully fed mosquitoes were separated from unfed and partially fed mosquitoes and reared in cages until day 5 post feeding. Midguts were dissected and stored in trizol in −80°C. The feeding experiments were performed for a minimum of three times for both blood and infected blood feeding and a total of 200–300 midguts were collected over a period of time for each sample. Total RNA was isolated separately from each lot and finally pooled during RNA seq library preparation. The libraries were made following manufacturer's instruction. RNA sequencing was performed using Illumina platform. The Total RNA quality was verified using RNA 6000 Nano Kit (Agilent Technologies, USA) on 2100 Bioanalyzer (Agilent Technologies, USA), with a minimum RNA Integrity number (RIN) value of 7.

### Preparation of library and Sequencing

Three Paired-End RNA-seq libraries of *An. stephensi* were generated, one each from total RNA extracted from sugar fed mosquitoes (PVpSF), mosquitoes 5 days post blood feed, (PVpBF5D) and mosquitoes 5 days post infected blood feed (PVpiBF5D). The RNA-Seq library construction and sequencing was performed by commercial service providers (NxGenBio Life Sciences, New Delhi, India). Total RNA was used to enrich mRNA using Oligotex mRNA midi prep kit (QIAGEN, Germany). 2 µg of total RNA using oligo(dT) magnetic beads and fragmented into 200–500 bp using divalent cations at 94°C for 5 min. The cleaved RNA fragments were copied into first strand cDNA using SuperScript II reverse transcriptase (Life Technologies, Inc.) and random primers. Fragments were A-tailed and end repaired after second strand cDNA synthesis. The cDNA libraries were constructed for the samples using the TruSeq RNA Sample Preparation Kit (Illumina, Inc.) with alternate fragmentation method for generating 200–500 bp fragments, according to manufacturer's instructions. The Paired-End RNA-Seq libraries were diluted and sequenced using TruSeq SBS Kit V3 on HiSeq2000 (Illumina, San Diego, CA) for generating 2×100 bp sequencing reads.

### Transcriptome Assembly and Read Mapping

**Figure 1:**
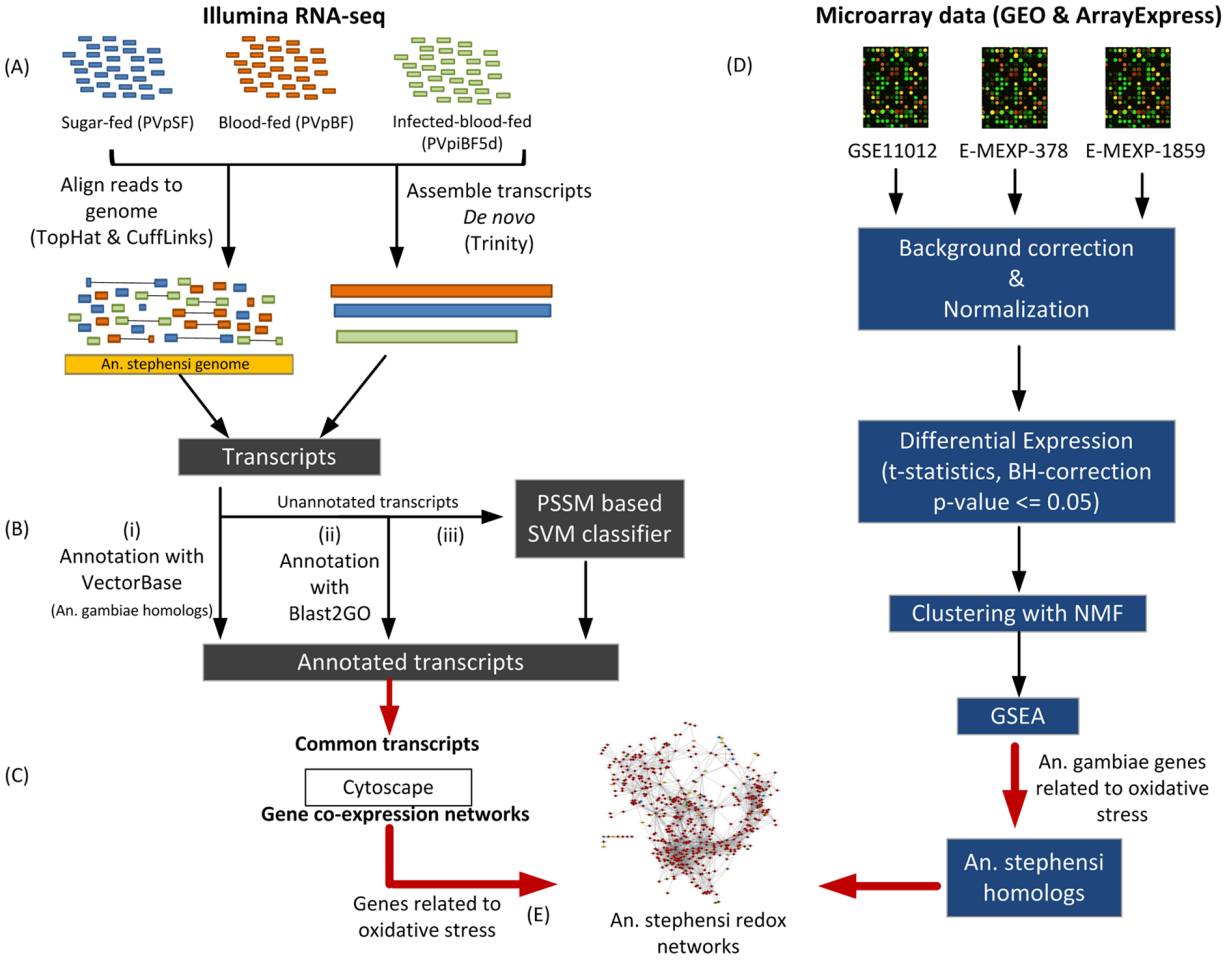
Pictorial representation of the workflow followed for the analysis of RNA-seq data and microarray data.

A simplified workflow of transcriptome assembly and analysis performed in this study is shown in Figure 1. The sequence reads of all the libraries were adapter trimmed using fastx toolkit and were subjected to quality check (QC) using FastQC retaining only high quality reads (>Q20) and discarding the rest [25]. The high quality reads were analyzed both by de novo assembly using Trinity [26] (data not shown) and by genome based analysis. TopHat [27] was used for genome mapping and *An. stephensi* genome was used as the reference genome which was downloaded from VectorBase [27]. Further analysis was performed with Cufflinks [27]. TopHat aligns the reads to the reference sequences using Bowtie tool [28] and realign the unaligned sequences by breaking them into small fragments.

### Functional annotation

One of the critical and essential steps in the analysis of high-throughput sequencing data is proper annotation of the assembled reads. The functional characterization of the assembled transcriptomes of all libraries of *An. stephensi* consisted of three steps (Figure 1). First, we performed homology based search of VectorBase mapped genes of *An. stephensi* against *An. gambiae*, a genome that is very well annotated and belongs to the same subgenus. We used BioMart tool from VectorBase to identify the homologs. Second, we used Blast2GO tool [29] to annotate the genes that were not annotated in the first step. Blast2GO blast the input sequence against NCBI nr database, retrieve the GO terms of the blast hits, assign the score to each GO term and finally select the lowest term from the branch of GO hierarchy tree to assign it to the input sequence. Third, we implemented support vector machine (SVM) to annotate the rest of the genes as described in next section.

### Annotation of genes from the transcriptome using Support Vector Machines

Evolutionary information can be extracted using Position Specific Substitution Matrix (PSSM), hence, for the functional characterization of the *An. stephensi* transcripts that were not annotated by simple Blast as described in the previous section; we built a predictive model based on PSI-BLAST (Position Specific Iterated BLAST). In order to build the predictive model we used SVM, a powerful supervised machine learning algorithm for prediction, that classifies an object into one or more classes based on the set of input feature vectors. In our study, PSSM generated by PSI-BLAST was used as the input feature vector for SVM. PSSM based classifiers has been reported as the most suited among the SVM classifiers [30]. PSSM generates 20xN matrix, where N is the length of the sequence of the query. To make input feature vectors of fixed length we normalized the matrix using logistic function.

**Figure 2:**
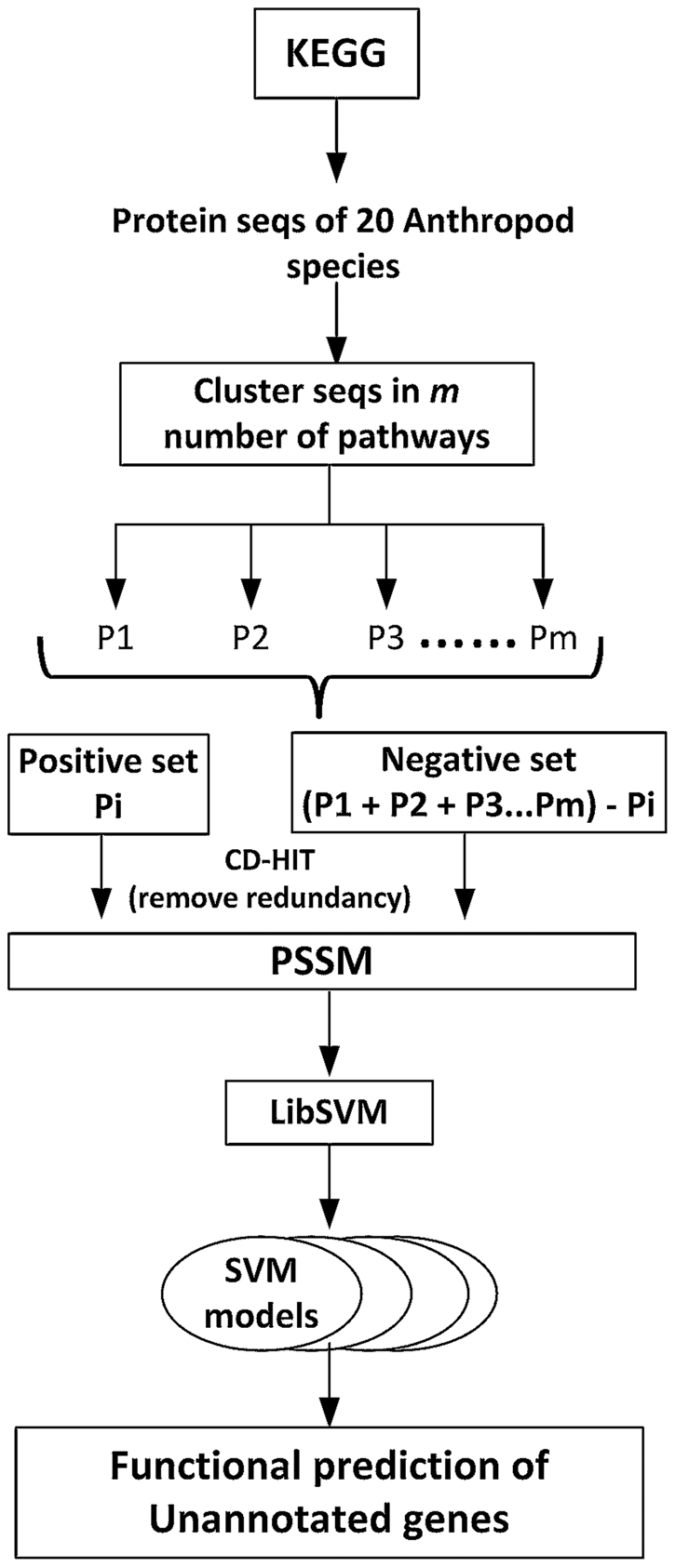
The figure represents the SVM work flow used in this study.

We built the training data for SVM model by downloading protein sequences of 20 Arthropod species from KEGG and all the sequences, irrespective of the species, were clustered according to the selected pathways. We built *m* number of SVM models for *m* pathways. The training data for each of the *i*th model consisted of protein sequences of *i*th pathway of interest as positive set and protein sequences of the *m-i* pathways as negative set (Figure 2). The redundancy of the positive and negative datasets was removed using CD-HIT [31]. The lowest possible threshold for identity by CD-HIT was 40%; we used this threshold to generate non-redundant training datasets. As a 40% threshold reduced the size of positive dataset quiet a lot and resulted in highly imbalance training datasets, we also generated positive datasets by utilizing thresholds of 50%, 60% and 70%. We used libSVM with radial basis function as the kernel to build SVM model. To handle the class imbalance problem we penalized positive dataset using weight parameter of svm-train. We performed 5-fold cross-validations to estimate the values of cost, gamma and weight parameters. The final training datasets for each SVM model and the selected parameter values are given in Table S3. All the unannotated genes were then subjected to the SVM analysis. The pathway predictions of the genes were performed on the basis of SVM prediction score (Fig. 2).

### Evaluation of prediction models

We evaluated the performance of our classifiers by calculating accuracy, Receiver operating characteristic (ROC) curve and Area under Curve (AUC). ROC is a plot of false positive rate (1-specificity) on x-axis and true positive rate (sensitivity) on y-axis. The plot depicts the trade-off between specificity and sensitivity. The mathematical representations of the expressions can be represented as:

Sensitivity =  (TP/TP+FN)×100

Specificity =  (TN/TN+FN)×100

Accuracy =  (TP+TN/TP+FN+TN+FP)×100

MCC = (TP×TN) – (FP×FN)/sq.root [(TP+FN)×(TN+FP)×(TP+FP)×(TN+FN)]

Where TP means True positive, TN means True Negative, FN means False Negative and FP means False Positive.

### Differential expression and enrichment analysis

The differential expression analyses of the libraries were performed with Bioconductor package edgeR. edgeR uses TMM (Trimmed mean of M values) approach for the normalization of read counts. We identified differentially expressed genes by comparing all the three libraries with each other i.e. PVpSF vs PVpBF5D, PVpSF vs PVpiBF5D and PVpBF5D vs PVpiBF5D. edgeR analysis were performed by taking disperson value of 0.1 and p value < = 0.05.

Differentially expressed genes from each comparison were taken separately and subjected to pathway enrichment analysis. For the identification of significantly enriched pathways the protein sequences of *An. stephensi* corresponding to differentially expressed genes were downloaded from VectorBase and were analyzed using KOBAS web server. Hypergeometric test was selected as statistical method and FDR correction was performed using Benjamin and Hochberg method (1995). Pathways with p< = 0.05 were considered as significant pathways.

### Microarray Data search

A data search was conducted to identify the relevant data sets to be used for the study. A search was performed using “Mosquito”, “*Plasmodium*”, “*Drosophila*”, “Oxidative stress” and “Infection” as key elements and wherever required, the related terms and alternative terms were also used. Online library and databases namely, PubMed, ArrayExpress and Gene Expression Omnibus (GEO) were searched for the data using the key words and the relevant data were downloaded (Table S1).

### Microarray Data Analysis

For the purpose of maximizing the information on redox dynamics of *Plasmodium* invasion in *An. stephensi*, we incorporated microarray experiment datasets with similar experimental setup from two other dipteran species, one of which belongs to the same subgenus i.e. *An gambiae* and the other is more closely linked by evolutionary and genetic lineage i.e. *Drosophila melanogaster*. We downloaded three datasets (Table S1) from public repositories Gene Expression Omnibus (GEO) and ArrayExpress namely (1) E-MEXP-378 : Transcription profiling of mosquitoes fed blood infected with two alternative *P. berghei* strains; wild type (wt) or an invasion-deficient, CTRP (Circumsporozoite- and TRAP-related protein) knockout (ko) strain (2) E-MEXP-1859: Transcription profiling of *Drosophila* transformed with two *Plasmodium* cell surface antigens, circumsporozoite protein (CSP) and Thrombospondin-related adhesive protein (TRAP) and (3) GSE11012: An analysis of the impact of infection by *Buchnera aphidicola* APS on gene expression of *Drosophila* S2 cells. All the three datasets were background corrected and normalized using LOESS and Aquantile normalization. The normalized data were further analyzed using Bioconductor packages LIMMA [32], [33]. We used eBayes function to calculate moderated paired t-statistics after fitting the linear model and assessed the genes expressing differentially using p-value cutoff of 0.05. The p-values were corrected for multiple testing with Benjamini and Hochberg's (BH). The differentially expressed genes were further clustered using Non-negative matrix factorization (NMF) [34], which were then subjected to pathway enrichment analysis using gene set enrichment analysis (GSEA) [35].

### Generation of *An. stephensi* gene-gene co-expression network

*An. stephensi* gene co-expression network was generated using ExpressionCorrelation plugin of Cytoscape (http://apps.cytoscape.org/apps/expressioncorrelation) [36]. ExpressionCorrelation uses Pearson Correlation Coefficient for computing similarity matrix. For generation of the significant network using expression values minimum of four datasets are required. For this purpose, in addition to the gene expression data of the three RNA-seq libraries in this study, namely, PVpSF, PVpBF5D, PVpiBF5D, one more RNA seq datasets, namely, PVxBF5D (generated from *An. stephensi* fed on human blood) was used to generate the gene co-expression network. Common genes among all the libraries, with their expression values were used as an input for generation of the gene co-expression network using ExpressionCorrelation plugin of Cytoscape. The identified and predicted genes related to oxidative stress were mapped onto the *An. stephensi* gene interaction network and a sub-network consisting of only these mapped genes was drawn out from the network.

## Results and Discussion

Integration of different types of genomic data provided new insights into the interactions that exist between genes that are otherwise not distinguishable while studying single data sets [37]–[39]. In the present study, we inferred gene-gene interaction network of oxidative stress in *An. stephensi* during *P. vinckei petteri* development by integrating datasets originating from Illumina RNA-seq technology and gene expression microarrays.

### *Anopheles stephensi* midgut transcriptome during blood feed and *P. vinckei petteri* infection

In order to understand the dynamics of infection especially during *P. vinckei petteri* oocyst development in the midgut, we obtained the transcriptome of the midgut during the time point of mature oocyst development in the midgut. Physiologically, at this stage, the oocysts are mature and were ready to invade the midgut with oocyst derived sporozoites which is ideal for our study purpose of studying the oxidative stress in *Anopheles* during *Plasmodium* invasion. Analysis of such a cellular state is likely to reveal metabolic homeostasis facilitating parasite maturation and most importantly minimal metabolic penalty on vector. We dissected the midguts at day 5 post infection and processed those midguts that had maximum oocysts [40]. Total RNA was isolated from the midgut of mosquitoes at different time points and at different conditions over several feeding experiments (minimum of three times). The RNA was extracted from each group independently and midguts of around 200–300 mosquitoes for each condition were finally pooled during library preparation and sequenced in one run due to budget constraints. A total of 1.28×10^8^ reads , which includes 4.21×10^7^ reads from PVpSF library, 4.24×10^7^ reads from PVpBF5D library and 4.37×10^7^ reads of PVpiBF5D library were further processed (Table 1). The quality scores of reads were assessed and reads were trimmed by keeping quality score threshold as 20. We got 86.45% reads of PVpSF, 85.64% reads of PVpBF5D and 89.26% reads of PVpiBF5D, which were subjected to de novo assembly (data not shown) as well as reference mapping and assembly using TopHat and Cufflinks. Reference mapping with the newly published *An. stephensi* genome identified a total of 10496 genes in PVpSF, 9974 genes in PVpBF5D and 9613 genes in PVpiBF5D libraries from a total of 13650 genes present in VectorBase.

**Table 1:** Mapping summary of RNA-seq data.

S. No.	Midgut Samples	Total number of reads	No. of reads after quality trimming	Total number of *Anopheles stephensi* genes
1.	Sugar-fed mosquito (5 dpi)	4.21×10^7^	3.64×10^7^	10496
2.	Blood-fed mosquito (5 dpi)	4.24×10^7^	3.63×10^7^	9974
3.	*P. vinckei petteri* infected blood-fed mosquito (5 dpi)	4.37×10^7^	3.90×10^7^	9613

*RNA-seq libraries were mapped to *Anopheles stephensi* genome using TopHat and Cufflinks tools.

### Diverse *An. stephensi* genes are impacted during *Plasmodium vinckei petteri* infection

**Figure 3:**
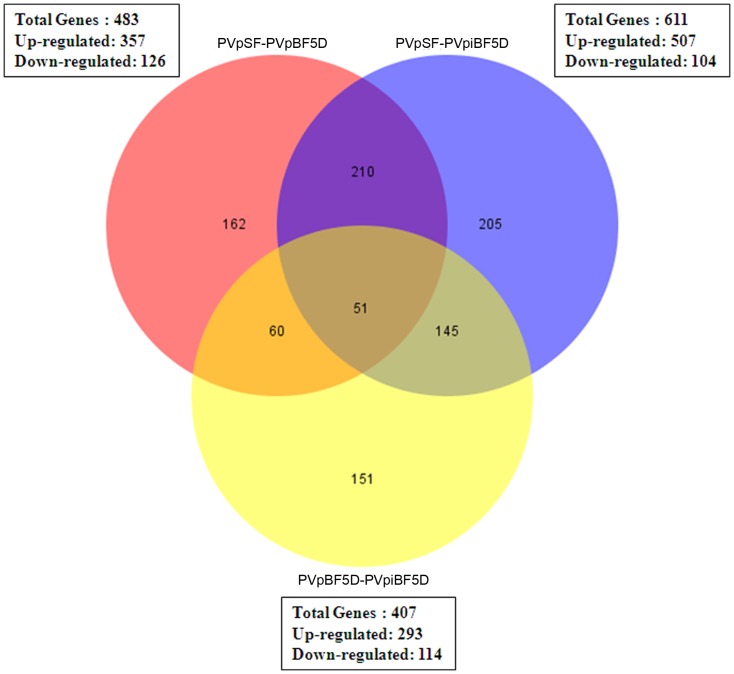
Venn diagram representing data summary of differentially expressed *Anopheles* genes from RNA-seq data.

To understand oxidative stress in *An. stephensi* during *P. vinckei petteri* infection, it is important to understand the regulation of the *An. stephensi* transcripts at this stage. For this purpose, we analyzed the expression pattern of the transcripts both at their relative abundance state and the consequence at the related impacted pathways. At the transcript level, within each library, on the basis of fold change in abundance, and P-value, a total 1501 genes were found to be differentially expressed in all the libraries taken together. Upon blood feeding, 483 genes were found to be differentially expressed, out of which 357 genes were found to be up regulated and 126 genes were down regulated. Upon parasitized blood feeding, 611 genes were differentially expressed out of which 507 genes were up regulated and 104 genes were down regulated. When compared between PVpiBF5D and PVpBF5D libraries that would emphasize on role of parasite development in the mosquito, 407 genes were found to be differentially expressed of which 293 genes to be up regulated and 114 genes were down-regulated. (Figure 3).

**Figure 4:**
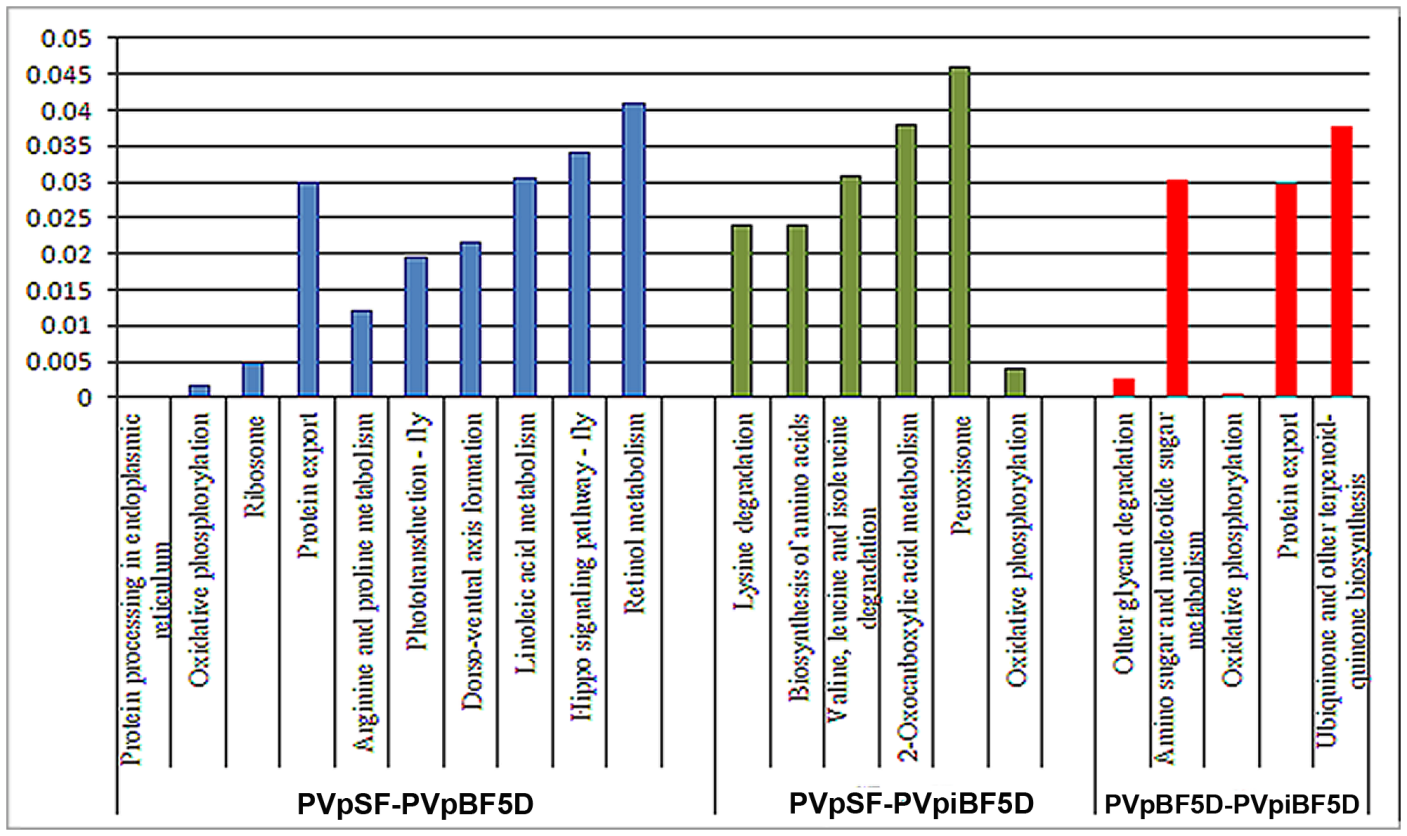
KOBAS analysis of differentially expressed genes. Graph represents the significant pathways predicted after KOBAS analysis.

Additionally, the differentially regulated transcripts of all the three libraries were analyzed for their pathway information. The transcripts were clustered into different pathways using KOBAS web server [41] (Fig. 4). Upon blood feeding, ten pathways were found to be significantly regulated (P value <0.05), four to be up-regulated and six found to be down-regulated. Upon parasitized blood feeding, it was found that out of six significant pathways, five pathways were down-regulated, with oxidative phosphorylation (OXPHOS) as the only pathway found to be up-regulated. When the infected blood fed and blood fed libraries were compared to see the impact of parasite development, it was seen that parasite development regulates five pathways significantly of which two were up-regulated and three were down-regulated. It is noteworthy that OXPHOS was up-regulated in both infected PVpSF5D vs PVpiBF5D and PVpiBF5D vs PVpBF5D libraries with high significance (p value <0.005), emphasizing the role of oxidative stress in *Anopheles* due to the *Plasmodium* development.

This interesting finding prompted us to further investigate those transcripts in these regulated OXPHOS pathways. We found a total of 20 genes to be impacted due to blood feeding and *Plasmodium* infection with most of the genes being part of the OXPHOS and the electron transport chain (Table 2). Previous studies have established the role of these important pathways in blood feeding in mosquitoes [42], [43]. Similarly, effects of *Plasmodium* infection in *Anopheles* OXPHOS have paved the way for better understanding of melanization in *Anopheles* [44]. Moreover, research has shown conserved nature of the OXPHOS genes within insects [45] and the importance and distribution of mitochondria in the midgut epithelia of mosquitoes [46], [47]. Recent studies have established the existence of dynamic mitochondrial supercomplexes on mammals, plants, yeast and bacteria [48]–[50]. These supercomplexes are categorized into five complexes based on their location and interaction with each other within the inner membrane of mitochondria [51]. In our transcriptome analysis, we observed that Complex I and IV were the most regulated during *Plasmodium* development while Complex III was also impacted upon blood feeding.

**Table 2:** The table shows the genes impacted due to blood feeding and *Plasmodium* infection.

Gene stable ID	Gene description	PVpSF	PVpBF5D	PVpiBF5D
ASTE000780	cytochrome c oxidase subunit 6b	239.485	64.1909	66.4754
ASTE001936	V-type H+-transporting ATPase S1 subunit	809.598	495.414	1122.98
ASTE002173	NADH dehydrogenase (ubiquinone) 1 alpha subcomplex 4	53473.4	62413.1	177530
ASTE003681	F-type H+-transporting ATPase subunit f	29635.9	35116.4	127126
ASTE004313	cytochrome c oxidase subunit VIIa	19671.2	16596.3	54387.4
ASTE004686	NADH dehydrogenase (ubiquinone) 1 beta subcomplex 4	704.049	378.93	877.12
ASTE006397	Mitochondrial cytochrome c oxidase subunit VIC	4825.3	1808.03	4017.93
ASTE006537	NADH dehydrogenase (ubiquinone) Fe-S protein 5	1672.32	551.183	1042.74
ASTE006599	V-type H+-transporting ATPase subunit G	5330.65	10551.3	20947.1
ASTE007332	Cytochrome b-c1 complex subunit 6, mitochondrial	2365.17	4372.13	15279.9
ASTE007457	cytochrome c oxidase subunit 6a, mitochrondrial	720.388	262.231	398.623
ASTE007459	NADH dehydrogenase (ubiquinone) 1 beta subcomplex 1	1149.47	2322.19	4313.28
ASTE007790	NADH dehydrogenase (ubiquinone) 1 alpha subcomplex 1	2571.73	755.536	530.959
ASTE007986	V-type H+-transporting ATPase subunit G	1152.51	2491.39	4094.67
ASTE009530	NADH dehydrogenase (ubiquinone) 1 alpha subcomplex 5	969.942	672.816	1840.72
ASTE009682	V-type H+-transporting ATPase subunit D	821.749	421.051	1207.4
ASTE010674	NADH dehydrogenase (ubiquinone) 1 subcomplex unknown 2	599.622	426.454	1739.31
ASTE011398	F-type H+-transporting ATPase subunit g	14815.4	22686.2	53015.7
ASTE011779	cytochrome c oxidase assembly protein subunit 17	180.156	319.84	489.181
ASTE014405	ubiquinol-cytochrome c reductase subunit 9	1604.72	95.9353	160.348

Most of the genes play role in the OXPHOS and the electron transport chain.

### *Anopheles* genes prediction in oxidative stress pathways using SVM

Comparative genomics can be used for the functional annotation of genomes that are not annotated completely. We annotated assembled transcriptome of the *An. stephensi* genome by identifying homologs of *An. gambiae* genes using VectorBase and Blast2GO tool. However, from a total of 13650 genes, 2516 genes were remained unannotated. We utilized PSSM based SVM classifier to annotate these genes and predicted putative genes that may be playing a role in the redox system of the mosquito during *Plasmodium* development. A total of 1352 non-redundant transcripts were classified into 8 different pathways according to their SVM scores (Table S3). The robust prediction performance of the SVM models is assured by ROC analysis (Figure S1). These genes were further utilized in generation of the oxidative stress network of *Anopheles*. The accuracy for Citric acid cycle was found to be ∼84%, PPP∼100%, Oxidative phosphorylation ∼98%, Jak ∼96%, MAPK ∼98%, Glycolysis ∼96% , TGF ∼96% and WNT ∼90%. The AUC (Area under Curve) values of these pathways supported the accuracy of the pathway models. The AUC value for the Glycolysis ∼0.9801, Citric acid cycle with AUC value of 0.8571, PPP with highest value 1, oxidative phosphorylation ∼0.9704, TGF ∼0.9978, WNT ∼0.944, for MAPK it is 0.9821 and for JAK is predicted to be 0.9762.

### Analysis of *An. stephensi* gene-gene co-expression network

In the last decade, importance of gene interaction network using integrated data sets in providing insights to gene functions and their interactions is evident from several studies [52]–[54]. The purpose of the study was to generate a network of the redox system of *An. stephensi* during *P. vinckei petteri* development and to understand the interactions of these participating genes during *Plasmodium* invasion of the midgut. For this purpose, it was important first to generate a broad interaction network of *An. stephensi* before segregating the redox related sub-set. For this purpose, we utilized the annotated genes of our transcriptome dataset to arrive upon a reference network on to which the oxidative stress genes identified and predicted in our study were mapped to infer the oxidative stress sub-network network of *An. stephensi* upon *P. vinckei petteri* infection with 516 nodes and 2904 edges (Fig. 4). The reference network was generated keeping default correlation threshold of −0.95 and 0.95. The final reference network was made up of 8871 non-redundant genes.

### Oxidative stress gene clusters using microarray data analysis

*Anopheles* data set E-MEXP-378 was an exhaustive microarray experiment involving time-points during the invasion of ookinete into the midgut of *Anopheles*. The original study was a functional genomic analysis of midgut epithelial responses in *Anopheles* during *Plasmodium* invasion [55] using a MMC1 (or 20 K) platform having total of 19,680 EST clones. The elegant experiment setup consisted of 53 sample data sets at three time points of ookinete invasion, namely 18–22 hrs post infection, 24–28 hrs post infection and 40–44 hrs post infection. A significant outcome of this study was the identification of remodeling/restructuring of the actin and microtubule cytoskeleton network due to *Plasmodium* infection. In the present study, we identified differentially expressed *An. stephensi* genes in oxidative stress during the early stages of *P. vinckei petteri* invasion.

Since *Anopheles gambiae* genome is poorly annotated, we extrapolated orthologs/paralogs of redox system from a *Drosophila* microarray dataset. A previous study has utilized the robustness of the *Drosophila* system to identify genes that regulate *Plasmodium* growth in the mosquito [56]. For our analysis, we utilized the dataset from E-MEXP-1859 which originally was a *Drosophila* dataset with Plasmodial genes knocked in to understand the function of two important *Plasmodium* invasion molecules [57]. The study used *Drosophila*, a model system, to understand the role of Plasmodial surface antigens in *Plasmodium* invasion. The authors were able to provide evidence of the role of immunity genes in this process using 21 chips of 13,614 *Drosophila* genes. In our study, in addition to identifying genes affected upon *Plasmodium* maturation, this dataset was selected as reference data to identify genes playing role in oxidative stress but not yet annotated in *Anopheles*.

Previous studies have highlighted the impact of bacterial infection on the redox status in mosquitoes [19]. In order to identify those redox genes that are specific to *Plasmodium* invasion, another *Drosophila* microarray dataset (GSE11012) involving bacterial infection was selected as a control [58]. The study was performed over different time points of bacterial infection using 13842 unique gene IDs. The genes common to bacterial infections were excluded from analysis.

For the purpose of identifying genes that may play a role in oxidative stress of *Anopheles* during *Plasmodium* development, detailed computational analysis was performed on the selected microarray datasets (see Materials and Methods). The entire probe Ids of the datasets were converted to their respective gene Ids separately, using an in-house Perl script. Cluster analysis using Non-negative matrix factorization (NMF) algorithm was performed those clusters with co-phenetic coefficient of 0.9611 and six clusters were selected for further analysis.

Gene Set Enrichment Analysis (GSEA) analysis of data E-MEXP-378 and E-MEXP-1859 revealed six pathways each having significant P-values and OXPHOS pathway was found to be common among them. GSEA results showed cytoskeleton organization and biogenesis, Wnt signaling pathway, JAK-STAT, p53 signaling pathway, pentose phosphate pathway and OXPHOS as significant pathways for E-MEXP-378 and likewise oxidative phosphorylation, response to oxidative stress, Toll pathway, response to ROS, melanization defense response and hydrogen peroxide catabolic response as significant pathways for E-MEXP-1859 dataset. The 13 common differentially expressed significant genes in dataset E-MEXP-1859 and GSE11012 were removed from the further analysis so as to reduce the false positive results. Detailed analysis of these datasets revealed 40 genes of *An. gambiae* (Table S2) and 145 genes of *D. melanogaster* to play a key role in oxidative stress response.

### Redox system of *Anopheles* is a complex network of gene interactions

For the purpose of inferring the redox system of *Anopheles*, a three prong approach was used. Our aim was to integrate different types of data sets and extract information for most of the genes that are playing role in oxidative stress in *Anopheles* including unannotated genes, information of genes in other insects, extract information available in public domain and our own experimental data. Previous studies performed by integrating such information have yielded much information in predicting gene function [59], [60].

**Figure 5:**
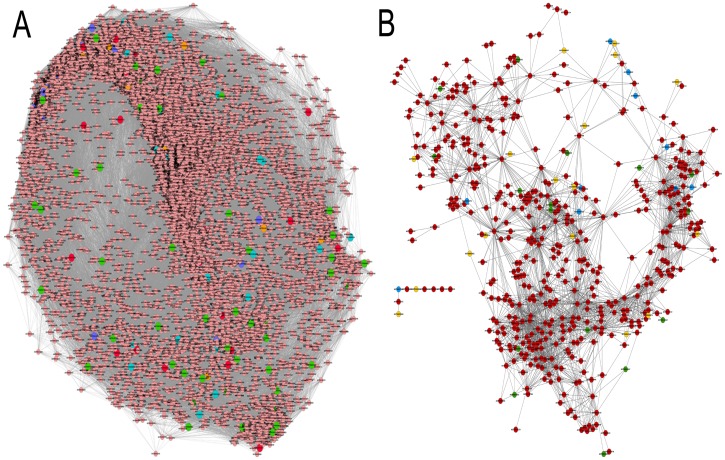
Gene interaction Co-expression network of *Anopheles stephensi*. (**a**) Meta-network showing interaction between the genes. The oxidative stress genes are highlighted in different colors. (**b**) A sub-network representing the interaction between oxidative stress related genes. Red color nodes shows genes predicted using SVM, Green color represents the GSEA predicted genes, Dark yellow node represent GO predicted genes, Light blue shows already annotated genes.

**Figure 6:**
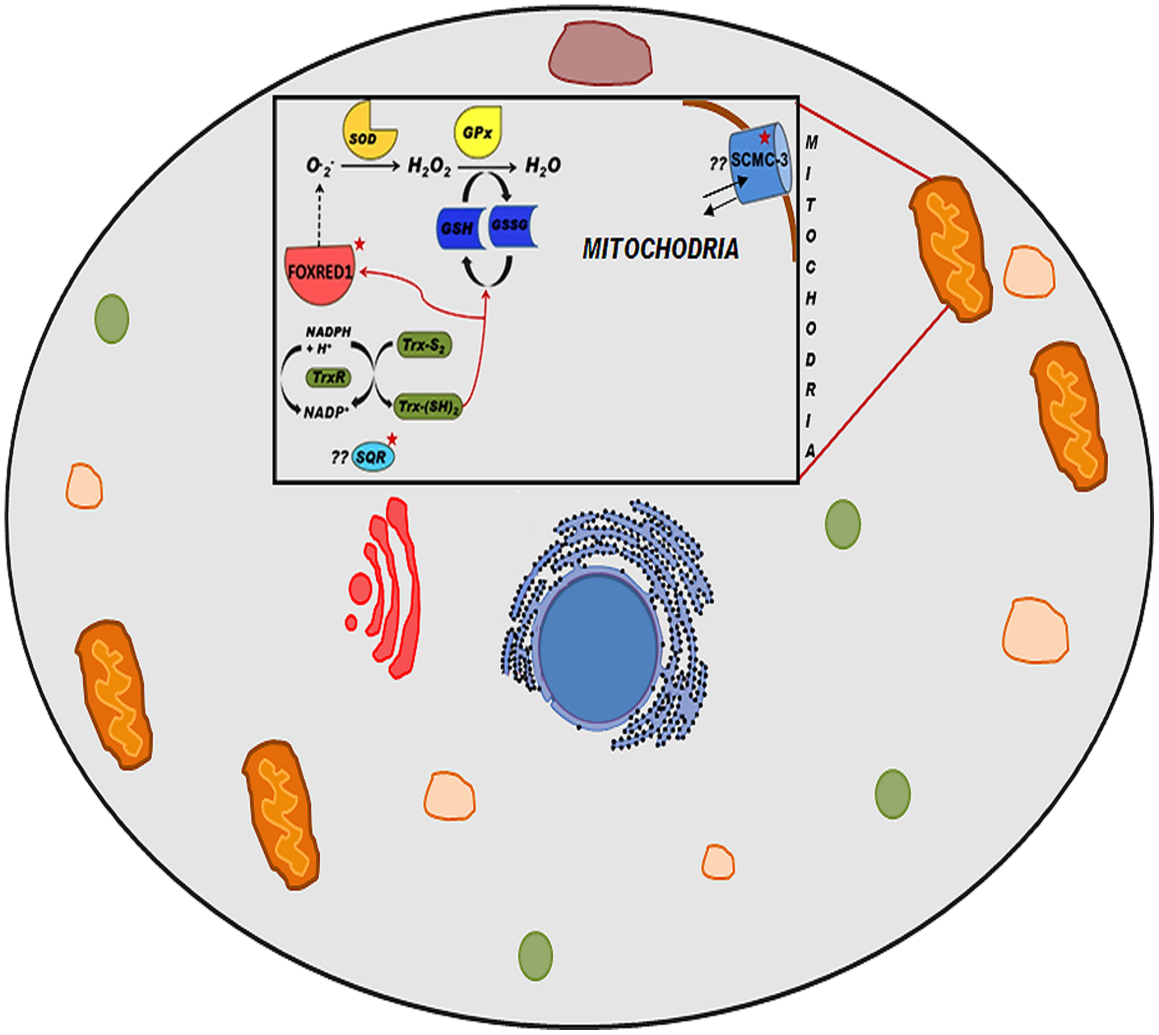
Figure representing the hypothetical model of oxidative stress pathway. This model includes 3 predicted proteins namely, FOXRED1, SCMC3 and SQR represented by red star.

The different methodologies finally resulted in a cluster of transcripts playing role in oxidative pathways (Table 3). Furthermore, those transcripts that were significantly regulated were mapped onto the reference network (Figure 5a) and a sub-network was extracted out of the meta-network (Figure 5b). Ookinete invasion of the midgut is triggered off by the adhesion of the parasite ookinete onto the epithelium of the mosquito midgut resulting in activation of processes that produce reactive oxygen species. Through our study including both de-novo study and reference mapping study using TopHat and Cufflinks, we hypothesize the involvement of some new molecules predicted by SVM and gene ontology in oxidative stress in *Anopheles* (Figure 6). Maintenance of ROS is accomplished by reduction of O2 to H2O while maximizing ATP synthesis [61] which is accomplished by the action of several enzymes like super oxide dismutase (SOD) and glutathione peroxidases. It is known that superoxide that is formed in mitochondria is produced by respiratory complexes and is detoxified by the action of the several species of SODs. Network generated in our study propose a possible role of FAD-dependent oxidoreductase domain-containing protein 1 (FOXRED1) in this conversion. It is known that FOXRED1 is localized in mitochondria and known to have chaperonic functionality in mitochondria complex 1 [62]. However, this has not been identified in midguts of mosquito prior to our study. Another possible involvement is that of Sulfide: Quinone oxidoreductase (SQR) in the reduction of thioredoxin in the thioredoxin-proxiredoxin pathway to limit accumulation of peroxides [63]. In addition to the above molecules, our study has proposed the role of small calcium binding mitochondrial carrier protein 3 (SCMC 3) in *Anopheles* redox homeostasis. Role of calcium in maintaining mitochondrial function and combating oxidative stress has been well documented [64].

**Table 3:** Expression pattern of the RNA-seq and microarray predicted genes related to oxidative stress.

Gene	Status	SF	BF5D	iBF5D
ASTE000971	Annotated	381.968	319.061	405.627
ASTE003004	Annotated	47.9869	40.0328	27.2383
ASTE003715	Annotated	27.2428	23.6363	13.0793
ASTE004575	Annotated	500.691	356.958	332.761
ASTE004646	Annotated	50.6197	42.9242	72.3875
ASTE006040	Annotated	12.7964	21.5695	31.2505
ASTE006069	Annotated	598.912	490.404	334.513
ASTE006760	Annotated	287.084	180.579	341.974
ASTE007589	Annotated	3.80819	4.85993	16.6953
ASTE008571	Annotated	68.1175	28.406	42.6152
ASTE009039	Annotated	0.423056	0.170206	0.229036
ASTE010206	Annotated	332.959	593.219	303.61
ASTE010699	Annotated	10.8378	8.66985	12.0489
ASTE010862	Annotated	26.6517	20.8814	22.1117
ASTE001492	Annotated;GO	4.81434	3.51005	5.44027
ASTE003100	Annotated;GO	686.938	848.5	1261.81
ASTE006711	Annotated;GO	174.985	203.972	182.272
ASTE008907	Annotated;GO	1812.43	1262.74	2702.9
ASTE009813	Annotated;GO	350.706	72.1614	73.289
ASTE010772	Annotated;GO	527.803	391.384	471.926
ASTE011022	Annotated;GO	3193.95	7203.98	22057.8
ASTE000131	GO	47.282	41.9047	96.9806
ASTE000143	GO	9.26815	5.86659	6.20112
ASTE000912	GO	86.6084	130.991	80.9358
ASTE001043	GO	210.914	102.15	147.858
ASTE001249	GO	153.995	102.33	201.927
ASTE001371	GO	128.209	100.369	85.1941
ASTE001567	GO	242.662	628.562	512.411
ASTE001773	GO	7351.09	5017.66	6439.2
ASTE002909	GO	115.25	121.663	32.7457
ASTE002914	GO	162.207	122.774	58.1257
ASTE002991	GO	13.0357	27.1839	34.4705
ASTE003073	GO	3.95207	5.35709	6.64083
ASTE003130	GO	111.493	70.7715	125.698
ASTE003223	GO	18.1754	14.4909	19.7527
ASTE003848	GO	3.05716	3.12325	2.94652
ASTE004135	GO	107.759	112.337	60.473
ASTE004515	GO	179.896	135.373	139.161
ASTE004690	GO	38.7047	30.4935	43.1306
ASTE004709	GO	4.87481	7.14552	13.1994
ASTE004710	GO	8.93586	10.5808	22.1294
ASTE005121	GO	3.3418	19.4879	20.4617
ASTE005165	GO	412.232	743.759	654.745
ASTE005387	GO	8.67074	55.0254	4.73109
ASTE005445	GO	251.465	152.927	107.231
ASTE005602	GO	371.038	314.707	204.804
ASTE005696	GO	77.6152	347.119	236.1
ASTE006080	GO	106.617	41.7707	47.5019
ASTE006277	GO	143.891	219.73	109.634
ASTE006361	GO	48.7165	43.3185	23.9857
ASTE006397	GO	4825.3	1808.03	4017.93
ASTE006501	GO	180.108	296.007	79.8115
ASTE007457	GO	720.388	262.231	398.623
ASTE007781	GO	138.289	138.146	182.986
ASTE007782	GO	144.385	111.405	62.9876
ASTE007973	GO	152.821	126.328	81.7444
ASTE008071	GO	161.366	90.8716	84.8784
ASTE008211	GO	3.89653	2.99567	6.48923
ASTE008276	GO	481.106	321.01	140.308
ASTE008687	GO	111.786	72.2075	115.313
ASTE008731	GO	37.1639	200.549	160.301
ASTE008825	GO	16.4813	30.7846	5.32786
ASTE008900	GO	99.0236	81.4236	78.6687
ASTE009014	GO	18.4728	16.4796	18.2949
ASTE009146	GO	217.069	185.006	178.595
ASTE009370	GO	86.3904	77.4245	120.952
ASTE009555	GO	24.3915	36.7647	33.2701
ASTE009860	GO	70.0946	49.8105	9.94767
ASTE010293	GO	343.966	322.758	399.022
ASTE010572	GO	26.2349	20.7426	17.9355
ASTE010775	GO	7.49661	7.50756	7.59294
ASTE011003	GO	11.0987	8.78333	5.66391

These genes were selected on the basis of GO terms, and microarray analysis. RPKM values of all the genes are also shown in the table.

*The analysis was performed using *Anopheles gambiae* genes. *Anopheles gambiae* IDs were then converted into *Anopheles stephensi* IDs using BioMart tool of VectorBase.

The cluster of genes involved in oxidative stress in *Anopheles* in our study is quite exhaustive. We have utilized both computational and high throughput data generation platforms to infer an almost complete redox system of *Anopheles* and proposed a model where new molecules could be playing important roles in oxidative stress of the malaria vector. Our proposed equilibrium awaits experimental validation.

## Supporting Information

Figure S1 **ROC plot showing the performance of the SVM models of different pathways.**
(TIF)

Table S1 **Table showing the data used in the study.**
(DOCX)

Table S2 **Table representing the GSEA predicted** ***Anopheles*** **genes (microarray data) which may play role in oxidative stress.**
(DOCX)

Table S3 **The excel sheet shows the SVM summary and the predicted SVM scores of unannotated transcripts.**
(XLS)
