# Supplementary figures and images for: Inference of the Oxidative Stress Network in Anopheles stephensi upon Plasmodium Infection
Source: PLoS One. 2014 Dec 4;9(12):e114461. doi: 10.1371/journal.pone.0114461 (PMC4256432; doi:10.1371/journal.pone.0114461)

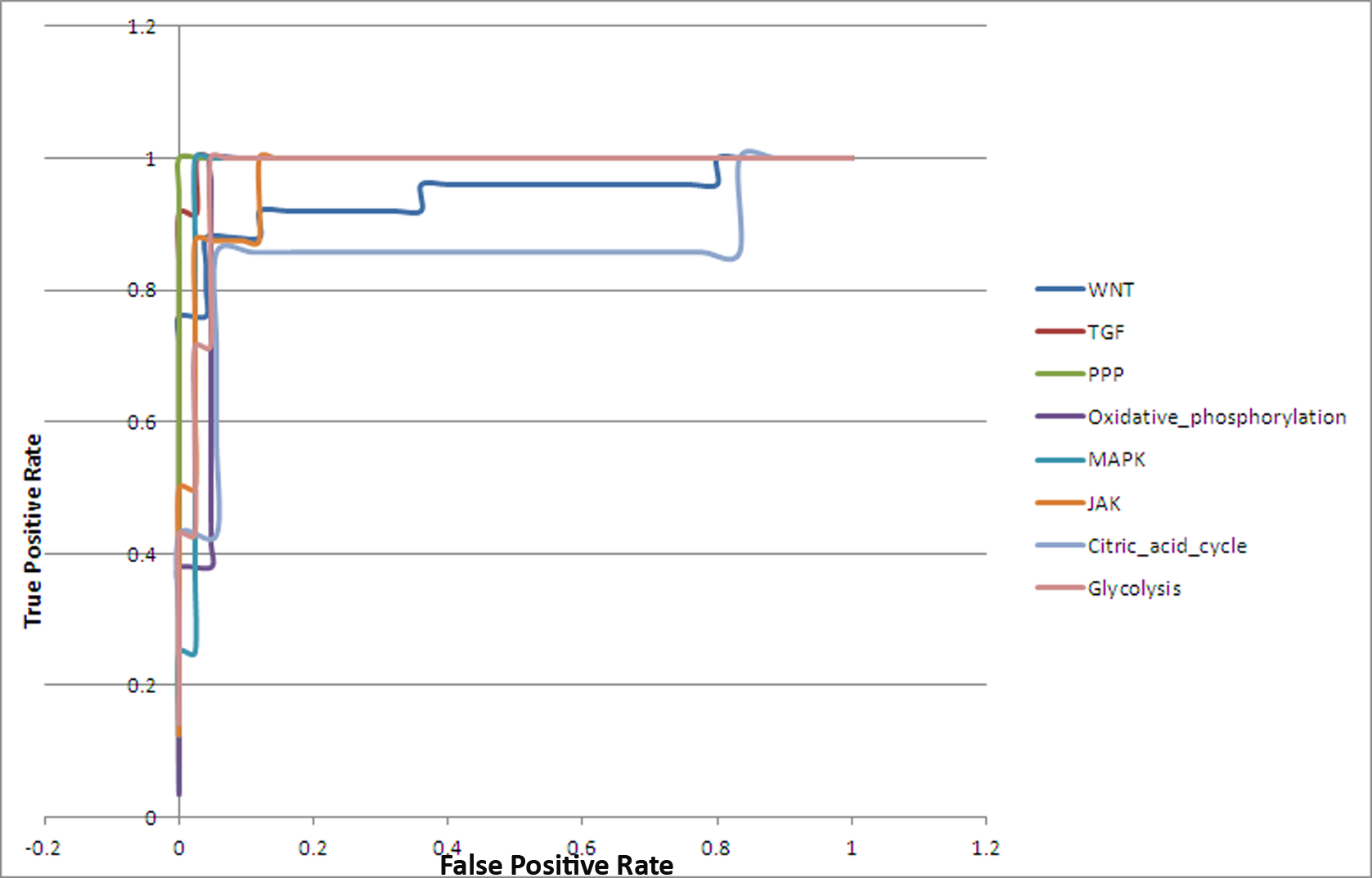

Supplement: Figure S1 — ROC plot showing the performance of the SVM models of different pathways. (TIF) [file pone.0114461.s001.tif]
