# Supplementary material for: Inference of the Oxidative Stress Network in Anopheles stephensi upon Plasmodium Infection
Source: PLoS One. 2014 Dec 4;9(12):e114461. doi: 10.1371/journal.pone.0114461 (PMC4256432; doi:10.1371/journal.pone.0114461)
Supplement: Table S1 — Table showing the data used in the study. (DOCX) [file pone.0114461.s002.docx]

**Table S1.** Table showing the data used in the study.

| S. No. | NAME | Accession number | Remarks |
| --- | --- | --- | --- |
| 1 | Transcription profiling of mosquitoes fed blood infected with two alternative P. berghei strains: wild type (wt) or an invasion-deficient, CTRP (C-ircumsporozoite- and TRAP-related protein) knockout (ko) strain | E-MEXP-378 |  |
| 2 | Transcription profiling of Drosophila transformed with two Plasmodium cell surface antigens | E-MEXP-1859 |  |
| 3 | An analysis of the impact of infection by Buchneraaphidicola APS on gene expression of Drosophila S2 cells | GSE11012 |  |
| 4 | RNA-Seq data of sugar fed Anopheles stephenshi mosquito | Submitted | In this study |
| 5 | RNA-Seq data of Blood fed Anopheles stephenshi mosquito after 5 days | Submitted | In this study |
| 6 | RNA-Seq data of Blood fed Anopheles stephenshi mosquito infected with Plasmodium vinckie | Submitted | In this study |
