# Supplementary material for: Inference of the Oxidative Stress Network in Anopheles stephensi upon Plasmodium Infection
Source: PLoS One. 2014 Dec 4;9(12):e114461. doi: 10.1371/journal.pone.0114461 (PMC4256432; doi:10.1371/journal.pone.0114461)
Supplement: Table S2 — Table representing the GSEA predicted Anopheles genes (microarray data) which may play role in oxidative stress. (DOCX) [file pone.0114461.s003.docx]

**Table S2.** Table representing the GSEA predicted *Anopheles* genes (microarray data) which may play role in oxidative stress.

| **Gene ID** | **Gene description** |
| --- | --- |
| AGAP004880 | L-lactate dehydrogenase |
| AGAP005031 | E3 SUMO-protein ligase PIAS2 |
| AGAP005134 | F-type H+-transporting ATPase subunit alpha |
| AGAP006383 | oligosaccharyltransferase complex subunit beta |
| AGAP006456 | NADH dehydrogenase (ubiquinone) Fe-S protein 3 |
| AGAP006569 | acetyl-CoA synthetase |
| AGAP007258 | aconitate hydratase 1 / homoaconitase |
| AGAP007642 | 6-phosphofructokinase 1 |
| AGAP007729 | activin receptor type-1 |
| AGAP001823 | V-type H+-transporting ATPase subunit G |
| AGAP002021 | lysophosphatidate acyltransferase |
| AGAP002192 | isocitrate dehydrogenase (NAD+) |
| AGAP002401 | V-type H+-transporting ATPase subunit E |
| AGAP002884 | V-type H+-transporting ATPase subunit B |
| AGAP002914 | galactokinase |
| AGAP003153 | V-type proton ATPase catalytic subunit A |
| AGAP003168 | isocitrate dehydrogenase |
| AGAP003398 | nucleosome-remodeling factor 38 kDa subunit |
| AGAP003405 | adenylosuccinate synthase |
| AGAP003652 | aldehyde dehydrogenase (NAD+) |
| AGAP004055 | 2-oxoglutarate dehydrogenase E2 component (dihydrolipoamide succinyltransferase) |
| AGAP004376 | aldose 1-epimerase |
| AGAP004437 | glycerol-3-phosphate dehydrogenase |
| AGAP001587 | V-type H+-transporting ATPase subunit I |
| AGAP000880 | suppressor of cytokine signaling, invertebrate |
| AGAP010549 | L-iduronidase |
| AGAP011161 | transketolase |
| AGAP011208 | hexokinase |
| AGAP011768 | growth factor receptor-binding protein 2 |
| AGAP011800 | transaldolase |
| AGAP008719 | S-phase kinase-associated protein 1 |
| AGAP009537 | cytochrome c |
| AGAP009623 | glyceraldehyde 3-phosphate dehydrogenase |
| AGAP009334 | V-type H+-transporting ATPase 21kDa proteolipid subunit |
| AGAP007852 | aconitate hydratase 1 / homoaconitase |
| AGAP010298 | V-type H+-transporting ATPase subunit D |
| AGAP009264 | ethanolamine-phosphate cytidylyltransferase |
| AGAP009161 | deoxyribose-phosphate aldolase |
